# Extending the CARE Principles: managing data for vulnerable communities in wartime and humanitarian crises

**DOI:** 10.1038/s41597-025-04756-9

**Published:** 2025-03-11

**Authors:** Yana Suchikova, Serhii Nazarovets

**Affiliations:** 1 Berdyansk State Pedagogical University, 71100 Zaporizhzhia, Ukraine; 2 Borys Grinchenko Kyiv Metropolitan University, 04053 Kyiv, Ukraine

**Keywords:** Ethics, Ethics

## Abstract

The CARE Principles (Collective Benefit, Authority to Control, Responsibility, Ethics) were developed to ensure ethical stewardship of Indigenous data. However, their adaptability makes them an ideal framework for managing data related to vulnerable populations affected by armed conflicts. This essay explores the application of CARE principles to wartime contexts, with a particular focus on internally displaced persons (IDPs) and civilians living under occupation. These groups face significant risks of data misuse, ranging from privacy violations to targeted repression. By adapting CARE, data governance can prioritize safety, dignity, and empowerment while ensuring that data serves the collective welfare of affected communities. Drawing on examples from Indigenous data governance, open science initiatives, and wartime humanitarian challenges, this essay argues for extending CARE principles beyond their original scope. Such an adaptation highlights CARE’s potential as a universal standard for addressing the ethical complexities of data management in humanitarian crises and conflict-affected environments.

## Introduction

The CARE Principles (Collective Benefit, Authority to Control, Responsibility, Ethics) have emerged as a cornerstone for ethically managing data related to Indigenous peoples^1^. Designed to complement the technical standards of FAIR (Findable, Accessible, Interoperable, Reusable), CARE emphasizes the cultural, ethical, and social dimensions of data stewardship. These principles ensure that data governance prioritizes community well-being, respects local values, and protects the rights of vulnerable groups. While initially focused on Indigenous data, CARE’s ethical imperatives can – and should – be extended to other vulnerable communities. This comment explores how the CARE Principles can be applied to data governance in conflict-affected environments, ensuring ethical stewardship of sensitive information for vulnerable populations. While the discussion is framed through the lens of the Russo-Ukrainian war – focusing on internally displaced persons (IDPs) and civilians in occupied territories – the insights presented here have broader implications for humanitarian data management in other war-affected regions. By doing so, CARE can offer a unified ethical framework for addressing the challenges of data governance in complex humanitarian contexts.

Existing approaches to data governance, such as the FAIR principles, emphasize findability, accessibility, interoperability, and reusability to enhance data sharing and reproducibility. However, these principles, as outlined in recent studies^2^, often require extensive curatorial efforts and may not fully address ethical concerns in conflict-affected areas. The Guidelines for Research Data Integrity (GRDI)^3^ further highlight the risks of data misinterpretation and the necessity of maintaining accuracy and transparency. Yet, neither FAIR nor GRDI explicitly account for the agency and rights of vulnerable populations over their data. This gap underscores the relevance of the CARE principles, which prioritize collective benefit, authority to control, responsibility, and ethics in data governance^4^. By integrating CARE principles into existing frameworks, humanitarian organizations can ensure that data governance aligns with both ethical and practical considerations in high-risk contexts.

### The vulnerabilities of war-affected populations

The Russo-Ukrainian war has displaced millions, creating one of the largest displacement crises in Europe since World War II. Simultaneously, many individuals remain in occupied territories under precarious conditions. Both groups face heightened vulnerabilities linked to the collection and misuse of their data. For IDPs, the collection of personal information to coordinate humanitarian aid often results in privacy risks, ranging from data breaches to unauthorized use for non-humanitarian purposes^5^.

For those in occupied territories, data misuse can result in repression or violence, especially if sensitive information falls into the hands of occupying authorities^6^. A striking example is the use of digital records during so-called ‘filtration procedures,’ where occupying forces systematically verify individuals’ online activity, employment records, and prior affiliations to identify individuals deemed disloyal or politically undesirable. Reports from human rights organizations and independent investigators have documented cases where accessing certain digital content or having past government ties has led to arbitrary detentions and forced disappearances^7^.

Therefore, managing such sensitive data requires stringent ethical safeguards. Recent research highlights the importance of anonymizing participants in conflict-affected regions and mandating the destruction of sensitive information post-project to prevent harm^8^. This approach reflects a growing recognition of the critical intersection between data ethics and human security in conflict zones.

### Adapting CARE to new contexts

The principles of CARE provide a flexible yet comprehensive ethical foundation that can be adapted to diverse contexts. Its emphasis on collective benefit ensures that data governance serves the well-being of communities, while authority to control empowers affected populations to govern their own data. Responsibility demands accountability from data stewards, and ethics ensures that data practices uphold the highest moral standards. These principles align closely with the needs of war-affected populations and can address critical gaps in existing data management practices.

#### Collective benefit

The principle of collective benefit ensures that data collection and use actively contribute to community well-being. For IDPs and residents of occupied territories, this could mean leveraging data to improve humanitarian assistance, facilitate family reunifications, and support long-term recovery efforts. Lessons from projects like FAIR Island demonstrate how aligning data governance with community-defined goals can amplify its social impact^9^. Similarly, applying CARE in the Russo-Ukrainian context ensures that the primary focus of data use is the welfare of affected populations, not external interests.

A poignant example of the potential collective benefit is the documentation of forcibly deported Ukrainian children. A recent report by the Humanitarian Research Lab identifies 314 children illegally transferred to Russia^10^, where they were subjected to forced re-education and naturalization, often separated from siblings and stripped of their Ukrainian identity. Such documentation is crucial for reuniting families, bringing these children back to Ukraine, and ensuring that these war crimes are meticulously recorded for international accountability. Ethically and securely collected data can serve as a powerful tool in exposing these atrocities. By integrating CARE principles into data governance, humanitarian organizations and researchers can ensure that collected data not only protects vulnerable populations, but also actively contributes to their empowerment and restitution.

#### Authority to control

This principle addresses the critical issue of data sovereignty. For Indigenous communities, CARE has empowered them to reclaim control over data historically misused by external entities^11^. In the context of war, authority to control ensures that IDPs and those in occupied territories maintain autonomy over their personal information.

A compelling example of this principle in practice can be seen in how Ukrainian universities displaced due to occupation manage their online presence^12^. To safeguard the identities of faculty members who remain in occupied territories but continue teaching online, Ukrainian universities have deliberately hidden their personal profiles from public access^13^. Similarly, class schedules are withheld from public access to prevent sensitive operational details from being exploited. These measures highlight the importance of secure, community-controlled access to sensitive data, ensuring that the safety and autonomy of individuals are prioritized in crisis conditions.

Digital tools that enable such protections, akin to innovations like TK Labels for Indigenous knowledge^14^, are vital in war contexts. By embedding the principle of authority to control into data governance, organizations can uphold the rights of vulnerable populations and safeguard them from potential harm, ensuring that data serves their interests rather than becoming a tool for persecutions.

#### Responsibility

Responsibility in data governance demands accountability and transparency from those collecting, managing, and using data. This is especially important in contexts of war, where mistrust of institutions is widespread. By adopting CARE, organizations managing data in the Russo-Ukrainian context would be compelled to minimize harm, ensure informed consent, and foster trust with affected communities. As Belarde-Lewis *et al*.^14^ highlight, responsibility includes building relationships with communities and ensuring that data practices align with their cultural and ethical values.

Responsibility in data governance must extend beyond the technical process of collection to actively fostering trust and ensuring inclusivity, particularly in regions impacted by war. As highlighted by Popova *et al*.^15^, universities in Ukraine are implementing policies and strategies aimed at encouraging young people to return and participate in rebuilding their communities. These efforts emphasize a holistic approach to trust-building and reintegration, focusing on documenting local needs and aspirations while fostering unity among displaced populations. By doing so, universities shape data policies that transcend mere information collection, promoting collective action and community resilience.

Applying this framework within the context of the Russo-Ukrainian war through the lens of the CARE Principles demonstrates how responsibility in data governance can be a catalyst for reconstructing societal structures and empowering affected communities. Universities play a crucial role in this process by managing a range of data, including student enrolment records, research archives, and institutional collaborations. These datasets support post-war recovery by facilitating the reintegration of displaced scholars, tracking educational disruptions, and preserving intellectual heritage amid conflict. For instance, academic records help internally displaced students resume their studies without bureaucratic obstacles, while research databases contribute to understanding long-term societal impacts of the war^12^.

#### Ethics

The ethical dimension of CARE emphasizes the paramount importance of safeguarding the safety and dignity of individuals. In war zones, this principle becomes indispensable. Mishandling sensitive data can expose vulnerable populations to significant risks, such as targeted violence or stigmatization^16^. The application of CARE ensures that data governance upholds rigorous ethical standards, actively protecting individuals from harm.

Wartime conditions give rise to new vulnerable groups^17^, including those forcibly separated from their families, individuals unable to evacuate conflict zones, and people with disabilities who face heightened challenges in accessing aid and resources. These groups require particular attention to ensure their inclusion and protection in data practices. Ethical data governance must address these complexities by developing strategies to ensure that the unique needs of all vulnerable populations are met while mitigating harm. By adhering to CARE principles, institutions can ethically navigate these challenges, ensuring that their data practices prioritize both immediate security and long-term recovery for these at-risk groups.

Recent developments in data stewardship have acknowledged the challenges of implementing FAIR principles in practice, particularly in complex, interdisciplinary environments. A study by Fraga-González *et al*.^2^ introduced a pragmatic “FAIR enough” approach, suggesting that incremental adoption of FAIR principles is more feasible than rigid adherence. While this model provides flexibility, it does not directly address ethical concerns regarding data sovereignty and potential risks associated with misuse of sensitive datasets. In occupied cities, open data initiatives must be carefully assessed against potential risks, as seemingly neutral datasets – such as economic indicators or migration statistics – can be repurposed for surveillance and repression. This reinforces the argument that CARE principles are essential in complementing FAIR, ensuring that ethical accountability is central to data governance^18^. As Plastun and Kozmenko^19^ highlight, the misrepresentation of stolen Ukrainian universities as Russian entities in international academic databases is not merely a technical oversight but a serious ethical failure. This erroneous attribution legitimizes the occupation, distorts the academic landscape, and allows occupying authorities to falsely claim the intellectual and institutional achievements of Ukrainian academia as their own. This is particularly critical in conflict settings, where data about internally displaced persons or vulnerable communities may be repurposed for non-humanitarian aims. In this regard, the CARE principles serve as a necessary complement to FAIR by ensuring that data governance prioritizes ethical accountability and community oversight.

### Broadening CARE’s relevance and implications for global data governance

Some may contend that the CARE Principles, rooted in Indigenous contexts, are less applicable to other vulnerable groups. However, their adaptability has already been demonstrated across a variety of settings, from localized data stewardship projects to open science initiatives^9,20^ Extending CARE to war-affected populations does not compromise their original intent; rather, it underscores their universality and relevance as a robust ethical framework.

The need to broaden the application of CARE is particularly pressing given the challenges posed by the global data economy. As conflicts and displacements become more frequent, vulnerable populations are often at risk of exploitation by systems that prioritize efficiency over ethics. For instance, in Ukraine, the drive to publish FAIR datasets – critical for transparency and scientific progress – can unintentionally expose individuals to harm if ethical considerations are overlooked. CARE ensures that data contributes to scientific advancements responsibly, balancing openness with the protection of human rights and the welfare of communities.

In Ukraine, initiatives like the 2022 Open Science Plan aim to increase transparency and competitiveness in research^21^. Metrics such as the number of FAIR datasets published have been integrated into national evaluation criteria for research institutions. While these goals reflect progress, they must be tempered by the ethical demands of wartime realities. Data concerning displaced populations, occupied territories, or sensitive areas of research should not simply adhere to standards of openness, but must also be governed with care to avoid unintended harm. CARE principles provide this crucial safeguard, ensuring a balanced approach that retains the benefits of FAIR while mitigating the risks of uncritical data sharing. It is important to note that FAIR does not inherently imply openness; rather, it promotes well-structured and machine-readable data that can be accessed under clearly defined conditions. Sensitive datasets, particularly in humanitarian contexts, can adhere to FAIR principles while remaining restricted to authorized stakeholders to prevent misuse^22^

The global significance of CARE lies in its capacity to establish a standardized ethical framework for data governance across a wide range of contexts. Vulnerabilities are not limited to Indigenous populations or those affected by war; they also encompass marginalized communities, climate refugees, and individuals whose sensitive data is at risk of exploitation in sectors such as health, migration, and education. Adopting CARE globally would provide a unified ethical foundation that complements FAIR, ensuring that data governance prioritizes humanity’s collective welfare. From mitigating biases in artificial intelligence to ensuring equitable access to digital resources, CARE principles offer a universal approach for embedding ethics into the rapidly evolving data-driven landscape.

Applying CARE to populations affected by war demonstrates its ability to address systemic vulnerabilities heightened by displacement, conflict, and the misuse of data. In such scenarios, CARE ensures that data governance is not solely about technical efficiency but also centres in justice, equity, and the empowerment of communities^14,23^.

The integration of FAIR and CARE principles provides a global standard that harmonizes openness with ethical responsibility, setting a transformative model for tackling future challenges. By embedding CARE into global data governance structures, we can build systems that not only protect and empower vulnerable populations but also uphold the ethical obligations of the scientific and data management communities.

### Comparative analysis of CARE, FAIR, and international humanitarian data standards

The ethical and legal landscape of data governance in humanitarian contexts is shaped by multiple frameworks, including the FAIR principles, the CARE principles, and international humanitarian data protection standards established by the International Committee of the Red Cross (ICRC) and the United Nations (UN). While each of these frameworks offers valuable guidelines, they exhibit significant differences in scope, objectives, and applicability to conflict-affected populations or humanitarian crises.

FAIR principles focus on the technical and infrastructural aspects of data reusability, ensuring that datasets are structured for long-term scientific utility^22^. However, they do not inherently provide guidance on ethical considerations or community control over sensitive data. Recent critiques^2^ highlight that FAIR implementation often entails substantial financial and administrative burdens, which may be impractical in humanitarian crises. Moreover, the Guidelines for Research Data Integrity^3^ emphasize the risks of data misinterpretation and advocate for strict methodological controls, yet they do not address power dynamics in data governance or the agency of vulnerable populations over their information.

In contrast, the CARE principles emphasize ethical imperatives, advocating for the collective benefit of data use and the prioritization of the rights of affected communities^4^. This aligns with the ICRC’s approach to personal data protection, which underscores the need for strict limitations on data collection and use in humanitarian operations^24^. Additionally, the ICRC’s *Handbook on data protection in humanitarian action*^25^ provides practical guidance on implementing data protection measures in crisis settings, particularly in relation to biometric data, cloud storage, and cross-border data transfers. The handbook highlights the risks of unauthorized access and repurposing of humanitarian data, reinforcing the necessity of ethical oversight mechanisms, such as those proposed under the CARE framework. Similarly, the UN *Personal Data Protection and Privacy Principles*^26^ emphasize the importance of data minimization, informed consent, and purpose limitation, ensuring that personal data is not repurposed in ways that could harm vulnerable populations.

Legal analyses further demonstrate gaps in current frameworks. Kelemen^27^ highlights the absence of explicit protections for digital data in international humanitarian law (IHL), noting that electronic records do not enjoy the same safeguards as physical objects. This legal ambiguity is particularly concerning given the increasing militarization of digital data^28^, where biometric databases and online records can become strategic assets in armed conflicts. Such concerns reinforce the argument that the FAIR framework alone is insufficient in conflict scenarios, and that the ethical and community-driven perspectives of CARE must be integrated into data governance protocols. A structured comparison of these frameworks is presented in Table 1, illustrating their key attributes and areas of overlap.

**Table 1:** Comparative analysis of CARE, FAIR, and international humanitarian data standards in conflict-affected contexts.

Principle/Framework	Main focus	Key strengths	Limitations in conflict contexts
FAIR	Findability, accessibility, interoperability, reusability	Facilitates scientific reproducibility and machine-readability	Lacks ethical and community-driven oversight
CARE	Collective benefit, authority to control, responsibility, ethics	Emphasizes ethical data use and community governance	Requires institutional commitment for implementation
ICRC rules (2019) & Handbook (2024)	Humanitarian data protection	Strict safeguards against data misuse in humanitarian operations; practical guidelines for handling biometric and cloud-stored data	Limited applicability outside humanitarian agencies
UN Privacy Principles (2018)	Data minimization, informed consent, purpose limitation	Provides a broad ethical framework for personal data governance	Lack of enforcement mechanisms in conflict zones
IHL (Buchan & Lubin, 2022; Kelemen, 2024)	Legal protections for data in armed conflicts	Addresses military misuse of personal data	Unclear legal status of digital data

## Conclusion

The CARE Principles provide a robust and adaptable framework for ethical data governance, emphasizing the rights and welfare of vulnerable populations. Their application to communities impacted by the Russo-Ukrainian war highlights their broader relevance and potential as a universal ethical standard. By incorporating CARE, data practices can avoid causing harm and instead serve as tools for fostering resilience, supporting recovery, and advancing justice. This approach honours the dignity of war-affected populations and sets a transformative precedent for managing data in a world increasingly shaped by interconnected crises and conflicts.

This analysis underscores the necessity of integrating ethical and community-driven frameworks, such as CARE, into existing humanitarian and scientific data governance models. While FAIR principles remain fundamental for ensuring data usability, their limitations in conflict settings highlight the importance of complementary frameworks that prioritize accountability and affected populations’ rights. By advocating for explicit recognition of CARE within international humanitarian data policies – including ICRC and UN guidelines – this study contributes to the ongoing discourse on responsible data stewardship in crisis contexts.

In light of these insights, we urge researchers, policymakers, and organizations to actively engage in a dialogue about integrating CARE principles into their data governance frameworks. Such adoption will not only advance scientific progress but also ensure that data management prioritizes the dignity, security, and empowerment of vulnerable communities around the globe. By embedding CARE into global practices, we can build a foundation for ethical and equitable data governance in a rapidly changing world.
